# Diabetic ketoacidosis and hyperglycaemic hyperosmolar syndrome in patients with cancer: A multicentre study

**DOI:** 10.1016/j.clinme.2024.100262

**Published:** 2024-11-09

**Authors:** Rabia K. Shahid, Qasem Haider, Sunil Yadav, Duc Le, Shahid Ahmed

**Affiliations:** a Department of Medicine, University of Saskatchewan, Saskatoon, Saskatchewan, Canada; b College of Medicine, University of Saskatchewan, Saskatoon, Saskatchewan, Canada; c Saskatchewan Cancer Agency, Saskatoon Cancer Center, Saskatoon, Saskatchewan, Canada

**Keywords:** Cancer, Diabetes mellitus, Diabetes complications, Cancer treatment, Diabetic ketoacidosis, Hyperglycaemia hyperosmolar syndrome, Hospital mortality, Advanced cancer, Chemotherapy, Immunotherapy

## Abstract

**Background:**

Diabetic ketoacidosis (DKA) and hyperglycaemic hyperosmolar syndrome (HHS) are life-threatening complications of diabetes mellitus. However, limited data about DKA and HHS are available in patients with cancer. The current study aimed to determine characteristics and outcomes of patients with cancer who were admitted with DKA/HHS in a mid-size Canadian city.

**Methods:**

Consecutive adult patients with an active cancer who were admitted with DKA or HHS from January 2008 to December 2020 in the city of Saskatoon, Saskatchewan, Canada were retrospectively evaluated. A univariate logistic regression analysis was performed to examine the correlation of various clinical variables with hospital mortality.

**Results:**

During the study period 6,555 patients with diabetes and cancer were admitted in one of the three tertiary care hospitals. Among them 33 (0.5 %) eligible patients with DKA or HHS with a median age of 60 years (range 36–94 years) were identified. In 36 % of patients, DKA or HHS was the presenting manifestation of newly diagnosed diabetes. Of all patients, 66 % developed DKA and 73 % had an advanced cancer. Overall, 52 % patients received a systemic cancer therapy prior to the admission, and 41 % received steroids. Ten (42 %) of 24 patients with an advanced cancer died, compared to none of the nine patients with an early-stage cancer (*p* = 0.032). No clinical factors significantly correlated with hospital mortality.

**Conclusions:**

Although DKA or HHS is uncommon in patients with diabetes and cancer, it is the manifestation of undiagnosed diabetes in about one-third of patients with cancer. It has been associated with high hospital mortality in patients with advanced cancer.

## Highlights

• This retrospective cohort study evaluated 33 consecutive hospitalized adult patients with an active cancer and diabetic ketoacidosis (DKA) or hyperglycemic hyperosmolar syndrome (HHS).
• Patients with active cancer and DKA/HSS were relatively older and most had an advanced cancer. In about one-third cancer patients, DKA or HHS was the presenting manifestation of new onset diabetes.
• Overall, 30 % patients died. Administration of a systemic cancer treatment within 30 days of admission was associated with an odd ratio of hospital mortality of 3.0 (0.62–14.8).
• It is important to recognize DKA and HHS early in symptomatic cancer patients undergoing treatment to promptly initiate appropriate care and avoid inferior outcomes.

## Graphical abstract

**Figure:**
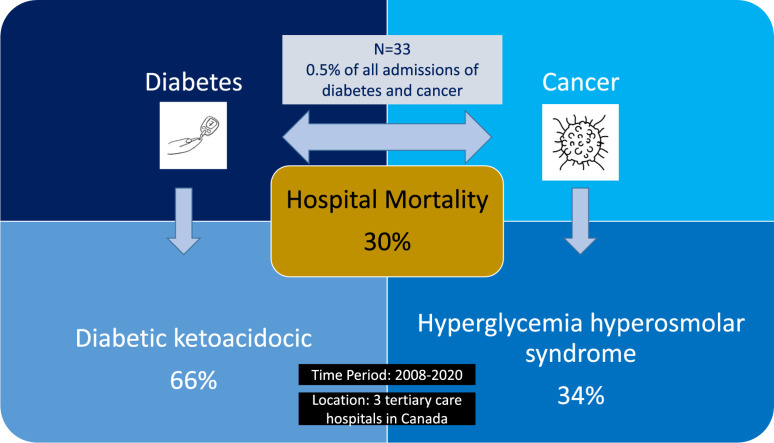

## Introduction

Despite a high prevalence of diabetes in patients with cancer, proportionally few studies have evaluated the impact of diabetes on cancer patient outcomes. About 10 % of the North American population have diabetes and, of them, approximately 95 % have type 2 diabetes.^1^^,^^2^ Diabetes has been associated with an increased risk of morbidity and mortality.^3^, ^4^, ^5^ Individuals with diabetes have an annual mortality twice that of the general population of similar age.^3^, ^4^, ^5^

About 20 % of patients with cancer have diabetes as a comorbid medical condition.^6^ Diabetes is not only a risk factor for several cancers, including cancer of the pancreas, liver, colon, breast and endometrium, but has also been associated with higher cancer-related mortality.^6^, ^7^, ^8^, ^9^ Patients with diabetes may have several underlying disease-related chronic complications including impaired kidney function, coronary artery disease, peripheral vascular disease, chronic infection and peripheral neuropathy among others, which may increase the risk of treatment-related complications.^6^^,^^7^ Several cancer therapies may induce diabetes, unmask underlying diabetes, or aggravate it.^6^^,^^10^^,^^11^ Furthermore, corticosteroids that are commonly used in the management of cancer are known to induce hyperglycaemia and may cause diabetes.^12^

Hyperglycaemic crises, including diabetic ketoacidosis (DKA) and hyperglycaemic hyperosmolar syndrome (HHS) are life-threatening complications of diabetes.^13^, ^14^, ^15^, ^16^ Patients with DKA have ketonaemia and metabolic acidosis.^13^^,^^14^ DKA is due to insulin deficiency with subsequent development of ketosis and metabolic acidosis. Some patients with cancer may present with diabetic ketoacidosis as the initial manifestation of underlying diabetes mellitus induced by underlying cancer or its treatment.^17^, ^18^, ^19^, ^20^, ^21^ A study showed that among non-oncological patients presenting with DKA, 47 % were known to have type 1 diabetes, 26 % were known to have type 2 diabetes and 27 % developed newly diagnosed diabetes.^13^ However, no such data are available in cancer patients. HSS, previously known as hyperosmolar non-ketotic diabetic coma typically occurs in individuals with type 2 diabetes. It is characterised by extreme hyperglycaemia, hyperosmolarity and dehydration without significant ketoacidosis.^22^ The incidence of HHS is lower than that of DKA. HHS accounts for less than 1 % of all diabetes-related hospital admissions, while DKA is more common, occurring in approximately 4–8 cases per 1,000 diabetic patients annually.^22^^,^^23^ A clinical overlap between DKA and HHS has been observed in over one-third of individuals experiencing hyperglycaemic crises.^16^

Of note, up to a third of individuals with cancer may have underlying undiagnosed diabetes. The stress of the newly diagnosed cancer and its treatment may unmask underlying diabetes in patients with cancer. Patients with cancer with underlying known or undiagnosed diabetes are at risk of developing DKA or HHS. The published literature on DKA and HHS in patients with cancer is mostly confined to case reports.^17^, ^18^, ^19^, ^20^, ^21^ The current study was undertaken in the context of scarce data on DKA and HHS in patients with cancer. It aimed to determine characteristics and outcomes of patients with cancer who were admitted with DKA or HHS in a mid-size Canadian city over a period of 13 years.

## Methods

### Study design and population

The study was approved by the University of Saskatchewan Biomedical Ethics Board (ID:2870). The University of Saskatchewan Ethics Board provided an exemption for informed consent. The study was performed in accordance with institutional research guidelines and regulations. In addition, operational approval for inpatient and outpatient data access was obtained from the Saskatchewan Health Authority and the Saskatchewan Cancer Agency, respectively. It was a retrospective cohort study of consecutive adult patients with cancer and DKA or HHS who were admitted to three tertiary hospitals in Saskatoon, SK, Canada from January 2008 to December 2020. The current metro area population of Saskatoon is 342,000 with a catchment area of about 0.5 million. Patients with a diagnosis of an active cancer and DKA or HHS during admission were eligible. The International Classification of Disease (ICD) code was used to identify eligible patients. All individual inpatient and outpatient oncology clinic medical records were reviewed by the study team. The study period was chosen, as individual inpatient records prior to 2008 were not available for review.

### Definitions

For the purposes of the study advanced disease was defined as follows: any stage IV solid cancer regardless of resection of metastases, a high-grade central nervous glioma, acute myelogenous or lymphoblastic leukaemia, high-grade lymphoma and multiple myeloma. Systemic therapy was defined as the use of oral or intravenous cytotoxic drugs, targeted therapy or immunotherapy. The diagnosis of DKA or HHS was recorded as per the medical team discharge diagnosis. For moderately to highly emetogenic chemotherapy, dexamethasone was commonly prescribed as a key component of the standard anti-emetic regimen. For most cases the dosing used was 8–12 mg prior to chemotherapy infusion, followed by 4–8 mg once daily on day 2, and 4–8 mg twice daily on days 3 and 4, with a total dose of 28–52 mg per cycle.

### Statistical analysis

For analysis patients were grouped into those with a previous history of diabetes and those with new-onset diabetes mellitus. Descriptive statistics were used to summarise the studied population demographics and baseline characteristics. Categorical data was summarized as frequency and its corresponding proportion. The Fisher exact test and Student's *t*-test were performed for analysis of categorical and continuous variables, respectively. For patients with more than one admission, information on first admission was used for data analysis and subsequent admissions were excluded.

A univariate logistic regression analysis was performed to identify the correlation between hospital mortality and various clinical variables. The following clinical variables were examined to assess their association with hospital mortality: age, sex, comorbid illness, advanced disease, DKA or HHS, gastrointestinal malignancy, systemic treatment in 30 days prior to admission, baseline hyperglycaemia, acidosis, white blood cell count, neutrophil and lymphocyte ratio, serum albumin, infection and the use of steroids 30 days prior to admission. SPSS version 28 (IBM, New York) was used for statistical analysis. The overall significance level was set at two-tailed p value of 0.05.

## Results

During the study period 6,555 patients with diabetes and cancer were admitted in one of the three tertiary care hospitals in the city of Saskatoon. Among them a total of 33 eligible patients were identified who were admitted with DKA or HSS. Their median age was 60 years (range 36–94 years), 48 % were men and 95 % had a comorbid illness. Among all patients, 21 (64 %) patients had a previous history of diabetes and 12 (36 %) patients developed new-onset diabetes mellitus. Among the 21 patients with a previous history of diabetes mellitus, seven (33 %) had type 1 diabetes, 10 (48 %) had type 2 diabetes and in four (19 %) patients it was not reported. Patient characteristics are presented in Table 1. Of 33 patients, 22 (66 %) had a diagnosis of DKA and 11 (34 %) presented with HSS. Of all patients, their mean glucose on admission was 32 ± 14.3 mmol/L, mean anion gap was 23 ± 11.7, mean arterial blood pH was 7.24 ± 0.10 and mean serum bicarbonate was 17 ± 7.2 mmol/L. Of 33 patients 29 (88 %) had a solid organ cancer. Among them gastrointestinal cancer was the most common (Table 2). Twenty-four patients (73 %) had advanced cancer. Overall, 17 (52 %) patients received systemic therapy, two (6 %) patients had radiation and one (3 %) had abdominal-perineal surgery for an ano-rectal tumour within 30 days of development of DKA/HHS. Patients with new-onset diabetes had a significantly high rate of systemic therapy and use of steroids (Table 1). Apart from these variables, no significant differences were noted between patients with a previous history of diabetes versus patients with new-onset diabetes mellitus.

**Table 1:** Baseline characteristics of patients with cancer and DKA/HHS.

Variable	All *N* = 33 (%)	Previous diabetes *N* = 21 (%)	Newly diagnosed diabetes *N* = 12 (%)	p-values
Median age	60 years (range: 36–94)	59 (36–94)	61 (46–74)	0.70
Age ≥70 years	10 (30)	8 (38)	2 (17)	0.25
Male	16 (48)	11 (52)	5 (42)	0.72
Active smoking	6 (18)	2 (10)	4 (33)	0.15
Solid organ cancer	29 (88)	20 (95)	9 (75)	0.12
Advanced cancer	24 (73)	14 (67)	10 (83)	0.42
Diabetic ketoacidosis	22 (66)	13 (62)	9 (75)	0.70
Mean serum glucose mmol/L	32 ± 14.3	33 ± 13	30 ± 16	0.55
Mean anion gap	23 ± 12	22 ± 12	25 ± 11	0.46
Mean pH	7.24 ± 0.10	7.23 ± 0.13	7.25 ± 0.11	0.66
Mean bicarbonate meq/L	17 ± 7	18 ± 7	15 ± 8	0.32
Mean potassium meq/L	4.8 ± 1.3	5.1 ± 1.3	4.4 ± 1.2	0.15
Mean sodium meq/L	134 ± 9	134 ± 11	135 ± 7	0.84
Mean creatinine umol/L	103 ± 52	108 ± 50	93 ± 56	0.44
Mean albumin g/L	28 ± 7	30 ± 7	27 ± 6	0.43
Mean alkaline phosphatase U/L	277 ± 323	315 ± 380	207 ± 176	0.45
Mean white blood cell count 10^9^/L	14.7 ± 15.2	18.5 ± 18.2	8.2 ± 4.8	0.08
Mean haemoglobin g/L	121 ± 24	117 ± 22	128 ± 28	0.24
Mean platelets 10^9^/L	261 ± 156	270 ± 136	245 ± 191	0.68
Mean neutrophil: lymphocyte ratio	13.1 ± 13.8	15.4 ± 16.6	9.1 ± 5.3	0.23
Recent cancer therapy	17 (52)	7 (33)	10 (83)	0.01
Received steroid	13/32 (41)	5/20 (25)	8 (67)	0.03
Infection	7 (21)	4 (19)	3 (25)	0.68
Death	10 (30)	5 (24)	5 (42)	0.43

**Table 2:** Distribution of cancer among patients with DKA/HHS.

Sites	Number
Gastrointestinal cancer^a^	9
Lung cancer	4
Haematological cancer^b^	4
Central nervous system tumours^c^	4
Genitourinary cancer^d^	3
Gynecological cancer^e^	2
Breast cancer	2
Unknown primary	2
Head and neck cancer^f^	2
Soft tissue sarcoma	1

a Colorectal cancer=4, gastro-oesophageal cancer=2, pancreatic cancer=1, bile duct cancer=1.

b acute leukaemia=2, chronic lymphocytic leukaemia=1, multiple myeloma=1.

c glioblastoma multiforme=3, low grade glioma=1.

d prostate cancer=1, kidney cancer=1, urinary bladder cancer=1.

e cervical cancer=1, ovarian cancer=1.

f tonsil=1, thyroid=1.

With respect to patients with DKA and HHS, no significant differences were noted between the two groups with respect to age, sex, stage of the disease or the use of active systemic therapy (Supplemental Table 1). Patients with HHS had a significantly high baseline creatinine whereas patients with DKA had a significantly low pH, HCO_3_, and elevated anion gap (*p* < 0.001). Patients with DKA had a significantly high neutrophil lymphocyte ratio compared to patients with HHS (73 vs 18 %, *p* = 0.008). Among 10 patients with type 2 diabetes, four developed DKA and the remainder experienced HHS.

Median duration of hospital stay was 8 days (range 2–63 days). Among triggers for DKA or HHS, 17 (52 %) received cancer therapy prior to admission, seven (21 %) had an infection, and seven (33 %) of 21 patients with a previous history of diabetes missed their medication. Of 17 patients who received systemic cancer treatment, 13 received chemotherapy, two received targeted therapy, and two patients received an immune checkpoint inhibitor, a PD-L1 inhibitor, with or without an anti-CTLA antibody (Table 3). In one of the two patients who received immunotherapy, diabetes was induced by an immune checkpoint inhibitor. Two patients were readmitted with recurrent DKA. Both survived their subsequent hospital admission.

**Table 3:** Systemic therapy within 30 days of DKA/HHS.

Sites	Number
FOLFOX	4
Carboplatin and paclitaxel	3
Temozolomide	2
FOLFIRI and bevacizumab	1
Cisplatin and gemcitabine	1
Pembrolizumab	1
Nivolumab plus ipilimumab	1
Lenalidomide and dexamethasone	1
Topotecan	1
Docetaxel and cyclophosphamide	1
Cytarabine and daunorubicin	1

FOLFOX=infusional 5FU and leucovorin and oxaliplatin.

FOLFIRI= infusional 5FU and leucovorin and irinotecan.

Of 33 patients, 10 (30 %) patients died. Five of 12 patients (42 %) with a newly diagnosed diabetes mellitus compared to five (24 %) of 21 patients with existing diabetes mellitus died during their hospital admission (*p* = 0.43). Ten (42 %) of 24 patients with an advanced cancer died compared to none of the nine patients with an early-stage cancer (*p* = 0.032). On logistic regression analysis, systemic cancer therapy with 30 days of admission (odds ratio [OR] for mortality: 3.0 [95 % confidence interval: 0.62–14.8]), new-onset diabetes mellitus (OR: 2.3 [0.49–10.5]), age ≥70 years (OR: 2.1 [0.36–12.5]) and infection (OR: 2.0 [0.36–11.48]) were associated with odds ratios of ≥2 for risk of hospital death; however, none were statistically significant (Table 4).

**Table 4:** Univariate logistic regression analysis and calculated odd ratios and 95 % confidence intervals of various clinical variables with hospital mortality.

Variables	Odd ratio (95 % CI)	*P*-values
Cancer systematic treatment within 30 days of admission	3.0 (0.62–14.8)	0.17
Newly diagnosed diabetes	2.3 (0.49–10.5)	0.28
Age ≥70 years	2.1 (0.36–12.5)	0.40
Infection	2.0 (0.36–11.48)	0.42
Men	1.95 (0.43–8.8)	0.38
Albumin < 35 g/L	1.95 (0.43–8.8)	0.38
Neutrophil: lymphocyte ratio >3.5	1.90 (0.18–19.5)	0.59
Leukocytosis	1.64 (0.36–7.38)	0.52
Hyperosmolar syndrome	1.52 (0.32–7.1)	0.59
pH<2.4	1.21 (0.24–6.27)	0.81
Use of steroid	1.03 (0.22–4.7)	0.96
Non-gastrointestinal cancer	1.02 (0.20–5.2)	0.98

Odd ratios for advanced disease and comorbid illness were not measurable as all who died had advanced disease and more than 95 % patients had a comorbid illness.

## Discussion

Our results showed that although DKA and HHS are relatively rare in patients with cancer, with a rate of five cases per 1,000 admissions, they were associated with a high mortality in such patients. Overall, about 30 % of patients died during admission. Furthermore, DKA or HHS was the presenting manifestation of underlying diabetes in more than 30 % of cancer patients. All patients who died during hospital admission had an underlying advanced cancer. Our results showed that DKA was more common than HHS, about two-thirds of patients with cancer developed DKA and one-third of patients experienced HHS. Patients with DKA or HHS require the timely identification of the underlying diagnosis with aggressive hydration and management of glucose and electrolytes along with treatment of underlying aggravating factors such as infection and intravenous insulin.^14^, ^15^, ^16^ Since symptoms of DKA or HHS may be masked by underlying cancer-related symptoms or treatment-related side effects or may be confused with cancer treatment-related side effects, it is important to keep a low threshold for DKA and HHS while managing cancer patients with nausea, vomiting, fatigue and other non-specific abdominal symptoms to avoid a delay in diagnosis of DKA or HHS and inferior outcomes. The risk of diabetes is especially high in certain cancers or specific targeted treatments. For example, diabetes is not only a risk factor for developing pancreatic cancer, but patients with pancreatic cancer also face a high risk of developing diabetes.^24^

Another important finding in our study is that the median age of the study population was 60 years. In the general population, DKA is more common in younger patients due to type 1 diabetes. For example, in a national sample of 220,340 patients admitted for DKA in the USA in 2017, 53.3 % were between the ages of 18 and 44 years. The mean age of these patients was 38.4 years.^25^ Clinicians should be aware that patients with cancer with known diabetes or a newly diagnosed diabetes are at risk of DKA, regardless of age.

A high rate of new-onset diabetes in patients with DKA and HHS and the fact that more than 80 % of patients with newly diagnosed diabetes developed it following the administration of systemic anticancer therapy in the study cohort signifies the importance of screening for diabetes in all patients with active cancer prior to embarking on systemic therapy. Patients with a high BMI or with a family history of diabetes or a personal history of gestational diabetes are at particularly high risk of development of diabetes during cancer treatment.^6^ Screening could include fasting or random blood glucose and haemoglobin A1c. Patients with an abnormal screening test will require a confirmatory test and management of their diabetes.

It is important to know that anticancer treatment could either induce insulin resistance or deplete insulin production by producing islet cell antibodies.^10^^,^^11^ New-onset type I diabetes is a known complication of immunotherapy and reports of insulin-dependent diabetes following anti-PD-1 alone or in combination with other anti-cancer agents have been published.^6^^,^^11^ In our study cohort, one patient developed type 1 diabetes and DKA following treatment of advanced non-small cell lung cancer with a single agent, pembrolizumab. In addition, cytotoxic agents can aggravate underlying diabetes and hyperglycaemia by causing dehydration from diarrhoea and decreased oral intake because of treatment-related anorexia, nausea and mucositis. The majority of patients in our cohort received combination chemotherapy that included a platinum compound, taxanes and 5FU. Of note, recent systemic therapy was associated with a 3-fold increased risk of hospital mortality; however, it was not statistically significant. As most patients with diabetes and cancer have other comorbid illnesses, multidisciplinary management of diabetic patients with cancer while they are on anticancer therapy with a focus on optimal blood glucose control, management of fluids and electrolyte balance, minimising cancer treatment-related gastrointestinal tract symptoms and reducing the risk of infection are important to avoid DKA and HHS and mortality.

In our study cohort, glucose lower medication omission was a triggering factor for DKA/HHS in about 33 % of patients with a known history of diabetes. These patients may have experienced decreased appetite, nausea, and gastrointestinal side effects from both their cancer therapies and glucose-lowering medications, which can impact their adherence to prescribed regimens. Ongoing patient education regarding adherence to glucose-lowering medication, home blood glucose monitoring, and self-management training is essential to reduce cancer treatment-related complications during active cancer treatment. In addition, about 40 % received steroids in combination with chemotherapy to reduce chemotherapy-related nausea, vomiting and fluid retention or to reduce tumour-related CNS oedema, intracranial pressure and thereby abnormal symptoms in patients with a CNS tumour. Patients on steroids are at about a two-fold higher risk of the development of diabetes.^6^ Hence, screening of diabetes and pre-diabetes along with optimal management of blood glucose in known diabetic patients are important in patients with cancer treated with corticosteroids. Patients on oral hypoglycaemic agents may require a short course of insulin or the addition of other glucose-lowering agents, while those with type I diabetes may need adjustment to their insulin regimen.

Insulin deficiency along with elevated counterregulatory hormone levels including growth hormone, cortisol, adrenaline, and noradrenaline results in DKA and HHS.^14^ It is important to note that while most cases of DKA are associated with type 1 diabetes, it is not uncommon in patients with type 2 diabetes. In our study cohort 40 % of patients with known type 2 diabetes developed DKA and 60 % experienced HHS. The pathophysiology of DKA in type 2 diabetes is different than in type 1 diabetes. Prolonged duration of type 2 diabetes mellitus can result in the loss of beta cell function and lead to DKA through mechanisms similar to those observed in type 1 diabetes mellitus. In addition, the relative suppression of insulin secretion, along with increased levels of circulating counterregulatory hormones and increased fatty acid levels, can contribute to the development of DKA in type 2 diabetes mellitus.^26^^,^^27^ Patients with DKA, compared to those with HHS, had a higher rate of leukocytosis, likely indicating an underlying inflammatory process. Although leukocytosis has been linked to worse outcomes in cancer patients, in this study, it did not correlate with inferior outcomes. This lack of association may be partially due to the small sample size.

To our knowledge, this is one of the largest reports on diabetes and DKA/HHS in cancer patients, spanning a period of 13 years. It is important to acknowledge several limitations of this study. In addition to its retrospective design and the reliance on medical records, which led to missing data on certain variables, such as the duration of diabetes, the diagnosis of DKA or HHS was based on hospital discharge records, which may introduce some classification bias. Furthermore, patients who developed DKA/HHS but did not have a diagnosis of DKA/HHS in their discharge summary could have been missed, resulting in underestimation of DKA/HHS rate in patients with cancer.

In summary, DKA and HSS are rare but potentially fatal complications of diabetes in patients with active cancer, and in about one-third of cases, they were the initial presentation of previously undiagnosed diabetes. These conditions are associated with high mortality, particularly in patients with advanced cancer. With approximately one in five cancer patients also having underlying diabetes, timely screening and optimal management are essential to prevent both acute and chronic complications. Type 2 diabetes mellitus increases the risk of various cancers, while advances in treatment have extended the lifespan of patients with type 1 diabetes, allowing them to reach ages where cancer diagnoses become more common. As the prevalence of both diabetes and cancer rises, and with the increasing use of newer glucose-lowering medications like sodium-glucose co-transporter-2 (SGLT2) inhibitors, which increase the risk of DKA, it is crucial to maintain awareness of these complications in cancer patients. Emerging diabetes technologies, such as continuous glucose monitoring (CGM) and hybrid closed-loop systems, may prove invaluable in preventing DKA by improving glucose control and enabling early detection of glycaemic abnormalities, offering a promising future for diabetes management in oncology care.

## Ethics approval and consent to participate

The University of Saskatchewan's Biomedical Research Ethic Board approved the study on 28 July 2021 (Application ID: 2870). The University of Saskatchewan Biomedical Research Ethics board provided an exemption for informed consent.

## Data availability statement

The data that support the findings of this study are not openly available due to reasons of sensitivity and will require approval from Biomedical Ethics Committee at the University of Saskatchewan, the Saskatchewan Cancer Agency and the Saskatchewan Health Authority. Data are located in controlled access data storage at the Saskatchewan Cancer Agency and the Saskatchewan Health Authority.

## CRediT authorship contribution statement

**Rabia K. Shahid:** Writing – review & editing, Writing – original draft, Supervision, Project administration, Methodology, Investigation, Formal analysis, Conceptualization. **Qasem Haider:** Writing – review & editing, Writing – original draft, Validation, Project administration, Investigation, Data curation. **Sunil Yadav:** Writing – review & editing, Project administration, Methodology, Investigation. **Duc Le:** Writing – review & editing, Writing – original draft, Methodology, Investigation. **Shahid Ahmed:** Writing – review & editing, Writing – original draft, Supervision, Project administration, Methodology, Investigation, Formal analysis, Conceptualization.

## Declaration of competing interest

No authors have any financial conflicts of interest in relation to the study to declare.

## Appendix Supplementary materials

Image, application 1
