# Supplementary material for: Diabetic ketoacidosis and hyperglycaemic hyperosmolar syndrome in patients with cancer: A multicentre study
Source: Clin Med (Lond). 2024 Nov 9;25(1):100262. doi: 10.1016/j.clinme.2024.100262 (PMC11635657; doi:10.1016/j.clinme.2024.100262)
Supplement: Supplementary file 1 [file mmc1.docx]

# Supplement

# Table 1: Baseline laboratory values of patients with cancer and diabetic ketoacidosis (DKA) or hyperosmolar hyperglycemic state (HHS)

|  |  |  |  |
| --- | --- | --- | --- |
| **Variable** | **DKA**  **N=22 (%)** | **HSS**  **N=11 (%)** | **P values** |
| Mean serum glucose mmol/l | 30.4±13.2 | 35.3±8.4 | 0.96 |
| Mean anion gap | 25.1±10.6 | 18.2±12.4 | <0.001 |
| Mean Ph | 7.22±0.13 | 7.29±0.19 | <0.001 |
| Mean bicarbonate  meq/l | 13±7.6 | 18.7±7.1 | <0.001 |
| Mean potassium    meq/l | 5.4±1.3 | 4.6±1.2 | 0.37 |
| Mean sodium        meq/l | 133±10.8 | 138±10.4 | 0.56 |
| Mean creatinine    umol/l | 90±56 | 112±50 | 0.006 |
| Mean albumin      g/l | 31±6.3 | 26±7.3 | 0.6 |
| Mean alkaline phosphatase U/l | 305±318 | 234±176 | 0.95 |
| Mean white blood cell count 10^9^/l | 17.5±18.2 | 9.2±4.8 | 0.03 |
| Mean hemoglobin        g/l | 118±22 | 126±28 | 0.06 |
| Mean platelets 10^9^/l | 275±136 | 255±191 | 0.9 |
| Mean neutrophil: lymphocyte ratio | 16.4 ±16.6 | 9.5±5.3 | 0.04 |
